# Supplementary material for: A large self-transmissible resistance plasmid from Nigeria contains genes that ameliorate a carrying cost
Source: Sci Rep. 2019 Dec 23;9:19624. doi: 10.1038/s41598-019-56064-z (PMC6927977; doi:10.1038/s41598-019-56064-z)
Supplement: Supplementary file 1 — Supplementary Information [file 41598_2019_56064_MOESM1_ESM.docx]

**A large self-transmissible resistance plasmid from Nigeria contains genes that ameliorate a carrying cost**

Rubén Monárrez, Molly Braun, Olivia Coburn-Flynn, João Botelho,

Babatunde W. Odetoyin, Jose I. Otero-Vera, Naa Kwarley Quartey,

Luísa Peixe, A. Oladipo Aboderin and Iruka N. Okeke

Supplementary Table 1. NCBI’s Conserved domains database (CDD) annotation of protein sequences from the pMB2 plasmid.

| **Query** | **Hit type** | **PSSM-ID** | **From** | **To** | **E-Value** | **Bitscore** | **Accession** | **Short name** | **Incomplete** | **Superfamily** |
| --- | --- | --- | --- | --- | --- | --- | --- | --- | --- | --- |
| pMB2_00004 | specific | 225853 | 1 | 214 | 1.78714e-71 | 217.006 | COG3316 | Rve | NA | cl26089 |
| pMB2_00005 | superfamily | 333150 | 1 | 266 | 0 | 535.011 | cl28330 | XerC superfamily | C | NA |
| pMB2_00006 | specific | 238127 | 45 | 197 | 7.52807e-52 | 163.848 | cd00209 | DHFR | NA | cl17279 |
| pMB2_00007 | superfamily | 331001 | 1 | 53 | 4.08942e-29 | 103.181 | cl26180 | DUF4111 superfamily | C | NA |
| pMB2_00008 | specific | 225853 | 1 | 214 | 1.78714e-71 | 217.006 | COG3316 | Rve | NA | cl26089 |
| pMB2_00009 | specific | 226950 | 7 | 254 | 4.95666e-68 | 213.087 | COG4584 | COG4584 | NA | cl27787 |
| pMB2_00010 | specific | 181681 | 1 | 259 | 0 | 501.543 | PRK09183 | PRK09183 | NA | cl26739 |
| pMB2_00011 | specific | 281099 | 2 | 122 | 9.74222e-50 | 161.261 | pfam03050 | DDE_Tnp_IS66 | N | cl24150 |
| pMB2_00011 | specific | 316346 | 128 | 164 | 1.2373e-14 | 63.8699 | pfam13817 | DDE_Tnp_IS66_C | NA | cl16419 |
| pMB2_00012 | specific | 313807 | 19 | 240 | 3.95703e-28 | 106.273 | pfam10670 | DUF4198 | NA | cl26499 |
| pMB2_00013 | superfamily | 331682 | 32 | 656 | 1.71037e-122 | 378.273 | cl26861 | CirA superfamily | NA | NA |
| pMB2_00014 | specific | 181833 | 1 | 121 | 2.44765e-72 | 211.2 | PRK09413 | PRK09413 | NA | cl21459 |
| pMB2_00017 | superfamily | 320824 | 44 | 217 | 3.00705e-23 | 91.9974 | cl00213 | DNA_BRE_C superfamily | NA | NA |
| pMB2_00018 | specific | 307271 | 11 | 134 | 0.00525809 | 37.2534 | pfam01051 | Rep_3 | C | cl19398 |
| pMB2_00019 | specific | 226950 | 1 | 110 | 9.22998e-08 | 48.2219 | COG4584 | COG4584 | C | cl27787 |
| pMB2_00020 | superfamily | 330231 | 2 | 113 | 4.67905e-68 | 210.007 | cl25410 | PolY superfamily | N | NA |
| pMB2_00021 | superfamily | 294470 | 2 | 115 | 1.11553e-70 | 207.13 | cl00721 | DDE_Tnp_IS1 superfamily | NA | NA |
| pMB2_00022 | specific | 238557 | 10 | 298 | 8.72155e-150 | 421.3 | cd01137 | PsaA | NA | cl00262 |
| pMB2_00023 | specific | 185016 | 1 | 272 | 0 | 526.373 | PRK15056 | PRK15056 | NA | cl28181 |
| pMB2_00024 | specific | 279318 | 9 | 265 | 2.59206e-77 | 235.2 | pfam00950 | ABC-3 | NA | cl00454 |
| pMB2_00025 | specific | 224033 | 1 | 263 | 2.72825e-68 | 212.782 | COG1108 | ZnuB | NA | cl27471 |
| pMB2_00026 | superfamily | 294470 | 2 | 115 | 1.11553e-70 | 207.13 | cl00721 | DDE_Tnp_IS1 superfamily | NA | NA |
| pMB2_00027 | superfamily | 307550 | 5 | 330 | 0.000420767 | 40.8216 | cl26263 | Viral_helicase1 superfamily | NA | NA |
| pMB2_00028 | specific | 173776 | 362 | 459 | 4.9365e-22 | 90.4186 | cd01026 | TOPRIM_OLD | NA | cl00718 |
| pMB2_00028 | superfamily | 331069 | 4 | 426 | 1.74672e-21 | 97.8578 | cl26248 | AAA_15 superfamily | C | NA |
| pMB2_00029 | superfamily | 326356 | 1 | 131 | 1.51174e-35 | 118.977 | cl14812 | PIN_SF superfamily | NA | NA |
| pMB2_00030 | superfamily | 321225 | 3 | 65 | 3.8342e-12 | 55.4626 | cl00877 | MazE_antitoxin superfamily | NA | NA |
| pMB2_00031 | specific | 184266 | 1 | 72 | 4.01511e-41 | 128.464 | PRK13710 | PRK13710 | NA | cl02188 |
| pMB2_00032 | superfamily | 321293 | 1 | 78 | 1.89068e-51 | 155.77 | cl00995 | PemK_toxin superfamily | C | NA |
| pMB2_00033 | specific | 278986 | 51 | 242 | 6.59536e-36 | 125.895 | pfam00589 | Phage_integrase | NA | cl00213 |
| pMB2_00035 | specific | 184293 | 1 | 245 | 2.31443e-166 | 459.035 | PRK13742 | PRK13742 | NA | cl19398 |
| pMB2_00036 | specific | 184261 | 1 | 388 | 0 | 836.939 | PRK13705 | PRK13705 | NA | cl27523 |
| pMB2_00037 | specific | 184254 | 1 | 323 | 0 | 604.15 | PRK13698 | PRK13698 | NA | cl26689 |
| pMB2_00038 | superfamily | 311089 | 16 | 139 | 1.90012e-40 | 137.139 | cl06126 | DUF1281 superfamily | NA | NA |
| pMB2_00040 | specific | 184255 | 1 | 227 | 0 | 529.021 | PRK13699 | PRK13699 | NA | cl17173 |
| pMB2_00042 | specific | 311216 | 1 | 136 | 9.78573e-72 | 211.22 | pfam07128 | DUF1380 | NA | cl08451 |
| pMB2_00044 | specific | 308711 | 44 | 141 | 1.80632e-35 | 117.697 | pfam03230 | Antirestrict | NA | cl03947 |
| pMB2_00045 | superfamily | 311216 | 1 | 98 | 3.97292e-07 | 45.9693 | cl08451 | DUF1380 superfamily | C | NA |
| pMB2_00049 | specific | 314891 | 90 | 209 | 9.75182e-43 | 146.635 | pfam12083 | DUF3560 | NA | cl13511 |
| pMB2_00050 | superfamily | 327401 | 30 | 95 | 8.80454e-05 | 39.896 | cl17173 | AdoMet_MTases superfamily | C | NA |
| pMB2_00051 | superfamily | 331561 | 9 | 62 | 0.000329615 | 36.8668 | cl26740 | MTS_N superfamily | N | NA |
| pMB2_00054 | specific | 184284 | 1 | 179 | 1.01249e-117 | 330.54 | PRK13732 | PRK13732 | NA | cl09930 |
| pMB2_00055 | superfamily | 310532 | 10 | 77 | 9.54189e-32 | 104.951 | cl05514 | DUF905 superfamily | NA | NA |
| pMB2_00056 | specific | 237477 | 1 | 144 | 7.37876e-101 | 285.101 | PRK13701 | psiB | NA | cl23984 |
| pMB2_00057 | specific | 184260 | 1 | 239 | 1.40685e-133 | 375.557 | PRK13704 | PRK13704 | NA | cl23935 |
| pMB2_00060 | specific | 310569 | 36 | 262 | 8.06949e-64 | 199.411 | pfam06067 | DUF932 | NA | cl12129 |
| pMB2_00061 | specific | 184274 | 1 | 169 | 2.10563e-124 | 346.683 | PRK13722 | PRK13722 | NA | cl00222 |
| pMB2_00062 | specific | 184269 | 8 | 125 | 6.99567e-67 | 197.584 | PRK13713 | PRK13713 | NA | cl23827 |
| pMB2_00063 | specific | 237479 | 1 | 228 | 2.48185e-93 | 272.375 | PRK13719 | PRK13719 | NA | cl28301 |
| pMB2_00064 | specific | 184292 | 1 | 126 | 1.46122e-29 | 101.782 | PRK13740 | PRK13740 | NA | cl27023 |
| pMB2_00064 | specific | 310249 | 70 | 118 | 3.99724e-18 | 71.9556 | pfam05509 | TraY | NA | cl27023 |
| pMB2_00065 | specific | 237485 | 1 | 121 | 4.50013e-56 | 170.109 | PRK13734 | PRK13734 | NA | cl11503 |
| pMB2_00066 | specific | 184263 | 1 | 103 | 8.21887e-62 | 182.911 | PRK13707 | PRK13707 | NA | cl06278 |
| pMB2_00067 | specific | 184278 | 1 | 188 | 3.84238e-133 | 370.259 | PRK13726 | PRK13726 | NA | cl05060 |
| pMB2_00068 | specific | 237486 | 1 | 242 | 3.71105e-143 | 400.249 | PRK13736 | PRK13736 | NA | cl05878 |
| pMB2_00069 | specific | 184281 | 1 | 475 | 0 | 864.519 | PRK13729 | PRK13729 | NA | cl26382 |
| pMB2_00070 | specific | 237488 | 1 | 196 | 6.47413e-126 | 352.636 | PRK13739 | PRK13739 | NA | cl11652 |
| pMB2_00071 | specific | 237482 | 1 | 65 | 9.27865e-34 | 109.266 | PRK13724 | PRK13724 | NA | cl23902 |
| pMB2_00072 | specific | 139817 | 1 | 83 | 1.9808e-52 | 157.988 | PRK13744 | PRK13744 | NA | cl10153 |
| pMB2_00073 | specific | 184285 | 1 | 171 | 2.70396e-92 | 265.487 | PRK13733 | PRK13733 | NA | cl19474 |
| pMB2_00074 | specific | 237480 | 31 | 874 | 0 | 1729.19 | PRK13721 | PRK13721 | NA | cl26286 |
| pMB2_00075 | specific | 184271 | 1 | 128 | 7.21596e-79 | 228.138 | PRK13717 | PRK13717 | NA | cl11515 |
| pMB2_00076 | specific | 184290 | 2 | 210 | 3.95934e-145 | 402.192 | PRK13738 | PRK13738 | NA | cl10149 |
| pMB2_00077 | specific | 237487 | 1 | 330 | 0 | 692.68 | PRK13737 | PRK13737 | NA | cl06067 |
| pMB2_00078 | specific | 184282 | 1 | 212 | 7.75224e-154 | 424.304 | PRK13730 | PRK13730 | NA | cl09883 |
| pMB2_00079 | specific | 237072 | 1 | 593 | 0 | 721.591 | PRK12355 | PRK12355 | NA | cl19475 |
| pMB2_00080 | specific | 172260 | 1 | 84 | 2.39617e-42 | 132.61 | PRK13718 | PRK13718 | NA | cl23903 |
| pMB2_00081 | specific | 184259 | 1 | 247 | 1.73174e-160 | 444.175 | PRK13703 | PRK13703 | NA | cl00388 |
| pMB2_00082 | specific | 184268 | 1 | 113 | 5.35883e-46 | 143.85 | PRK13712 | PRK13712 | NA | cl10138 |
| pMB2_00084 | specific | 237483 | 2 | 81 | 3.16209e-43 | 134.943 | PRK13727 | PRK13727 | NA | cl11516 |
| pMB2_00085 | specific | 237484 | 1 | 184 | 1.35683e-115 | 325.523 | PRK13728 | PRK13728 | NA | cl00388 |
| pMB2_00086 | specific | 184267 | 1 | 93 | 1.60326e-57 | 172.489 | PRK13711 | PRK13711 | NA | cl10137 |
| pMB2_00087 | specific | 184294 | 1 | 130 | 5.25104e-72 | 211.165 | PRK13743 | PRK13743 | NA | cl10152 |
| pMB2_00089 | specific | 184287 | 1 | 939 | 0 | 1771.97 | PRK13735 | PRK13735 | NA | cl26833 |
| pMB2_00090 | specific | 172283 | 1 | 169 | 1.128e-73 | 218.451 | PRK13741 | PRK13741 | NA | cl10151 |
| pMB2_00091 | specific | 184283 | 1 | 243 | 4.19184e-168 | 463.377 | PRK13731 | PRK13731 | NA | cl19728 |
| pMB2_00092 | specific | 184256 | 1 | 729 | 0 | 1594.61 | PRK13700 | PRK13700 | NA | cl26338 |
| pMB2_00093 | specific | 237478 | 2 | 1701 | 0 | 2975 | PRK13709 | PRK13709 | NA | cl26921 |
| pMB2_00094 | specific | 184262 | 20 | 267 | 3.33185e-169 | 467.144 | PRK13706 | PRK13706 | NA | cl05434 |
| pMB2_00095 | specific | 184304 | 1 | 185 | 1.93126e-112 | 317.581 | PRK13754 | PRK13754 | NA | cl26339 |
| pMB2_00097 | specific | 307599 | 88 | 160 | 3.03632e-15 | 67.7691 | pfam01527 | HTH_Tnp_1 | NA | cl21459 |
| pMB2_00098 | specific | 310376 | 9 | 107 | 4.90829e-57 | 171.479 | pfam05717 | TnpB_IS66 | NA | cl18171 |
| pMB2_00099 | specific | 281099 | 186 | 472 | 3.13267e-123 | 361.95 | pfam03050 | DDE_Tnp_IS66 | NA | cl24150 |
| pMB2_00099 | specific | 225970 | 105 | 262 | 9.8167e-35 | 127.563 | COG3436 | COG3436 | NA | cl26266 |
| pMB2_00099 | specific | 316346 | 479 | 515 | 5.71186e-14 | 65.4107 | pfam13817 | DDE_Tnp_IS66_C | NA | cl16419 |
| pMB2_00099 | specific | 315644 | 46 | 122 | 9.8774e-14 | 65.7784 | pfam13007 | LZ_Tnp_IS66 | NA | cl15234 |
| pMB2_00100 | superfamily | 294470 | 37 | 141 | 5.02737e-67 | 200.582 | cl00721 | DDE_Tnp_IS1 superfamily | C | NA |
| pMB2_00101 | specific | 307800 | 23 | 64 | 2.24625e-10 | 49.5654 | pfam01848 | HOK_GEF | NA | cl27487 |
| pMB2_00102 | specific | 184258 | 1 | 85 | 8.3074e-42 | 130.996 | PRK13702 | PRK13702 | NA | cl27844 |
| pMB2_00103 | superfamily | 299748 | 1 | 150 | 1.71336e-109 | 312.248 | cl11495 | IncFII_repA superfamily | C | NA |
| pMB2_00104 | specific | 234949 | 1 | 406 | 0 | 678.039 | PRK01388 | PRK01388 | NA | cl19186 |
| pMB2_00105 | specific | 183466 | 5 | 303 | 0 | 565.227 | PRK12354 | PRK12354 | NA | cl00452 |
| pMB2_00106 | specific | 179366 | 1 | 334 | 0 | 675.063 | PRK02102 | PRK02102 | NA | cl27385 |
| pMB2_00107 | specific | 224207 | 1 | 467 | 0 | 521.86 | COG1288 | YfcC | NA | cl21473 |
| pMB2_00108 | superfamily | 332404 | 9 | 155 | 1.66365e-47 | 150.842 | cl27583 | Arg_repressor superfamily | NA | NA |
| pMB2_00109 | specific | 310376 | 8 | 106 | 7.29209e-51 | 155.686 | pfam05717 | TnpB_IS66 | NA | cl18171 |
| pMB2_00110 | specific | 225511 | 1 | 112 | 2.01318e-08 | 48.3801 | COG2963 | InsE | NA | cl26146 |
| pMB2_00111 | superfamily | 294470 | 37 | 167 | 1.25516e-86 | 249.502 | cl00721 | DDE_Tnp_IS1 superfamily | NA | NA |
| pMB2_00112 | superfamily | 321016 | 26 | 135 | 1.08216e-23 | 89.6458 | cl00516 | Restriction_endonuclease_like superfamily | NA | NA |
| pMB2_00113 | specific | 315642 | 37 | 59 | 0.00994301 | 31.7873 | pfam13005 | zf-IS66 | N | cl26266 |
| pMB2_00114 | specific | 226077 | 4 | 328 | 4.38739e-54 | 179.355 | COG3547 | COG3547 | NA | cl27435 |
| pMB2_00115 | specific | 184258 | 1 | 84 | 1.60485e-41 | 130.225 | PRK13702 | PRK13702 | NA | cl27844 |
| pMB2_00116 | specific | 184300 | 1 | 285 | 0 | 542.597 | PRK13750 | PRK13750 | NA | cl11495 |
| pMB2_00117 | specific | 308235 | 121 | 207 | 1.93682e-09 | 52.5753 | pfam02517 | Abi | NA | cl00558 |
| pMB2_00117 | specific | 224185 | 42 | 216 | 0.000569969 | 39.5144 | COG1266 | YdiL | NA | cl27411 |
| pMB2_00118 | superfamily | 321225 | 6 | 83 | 1.57344e-11 | 54.2843 | cl00877 | MazE_antitoxin superfamily | NA | NA |
| pMB2_00119 | superfamily | 321293 | 2 | 109 | 1.54579e-38 | 124.951 | cl00995 | PemK_toxin superfamily | NA | NA |
| pMB2_00120 | specific | 307598 | 571 | 957 | 2.11434e-166 | 491.626 | pfam01526 | DDE_Tnp_Tn3 | NA | cl14901 |
| pMB2_00120 | specific | 316241 | 6 | 169 | 5.2268e-50 | 173.508 | pfam13700 | DUF4158 | NA | cl18663 |
| pMB2_00121 | specific | 306701 | 5 | 148 | 1.6107e-45 | 147.016 | pfam00239 | Resolvase | NA | cl02788 |
| pMB2_00121 | superfamily | 330857 | 127 | 200 | 0.00443407 | 36.4975 | cl26036 | YesN superfamily | N | NA |
| pMB2_00122 | superfamily | 326372 | 190 | 262 | 4.01875e-25 | 102.574 | cl14901 | DDE_Tnp_Tn3 superfamily | C | NA |
| pMB2_00123 | superfamily | 333100 | 18 | 273 | 1.90034e-40 | 140.907 | cl28280 | RhaT superfamily | NA | NA |
| pMB2_00124 | specific | 273318 | 46 | 184 | 1.76109e-28 | 108.506 | TIGR00880 | 2_A_01_02 | NA | cl21472 |
| pMB2_00124 | specific | 311564 | 10 | 352 | 2.14851e-23 | 99.8062 | pfam07690 | MFS_1 | NA | cl26865 |
| pMB2_00125 | superfamily | 331045 | 10 | 209 | 1.09714e-83 | 247.498 | cl26224 | TetR_C superfamily | NA | NA |
| pMB2_00126 | specific | 307598 | 101 | 487 | 0 | 587.155 | pfam01526 | DDE_Tnp_Tn3 | NA | cl14901 |
| pMB2_00127 | specific | 225853 | 1 | 214 | 1.78714e-71 | 217.006 | COG3316 | Rve | NA | cl26089 |
| pMB2_00128 | specific | 275224 | 16 | 199 | 3.24095e-142 | 393.481 | TIGR04431 | N6_acetyl_AAC6 | NA | cl26156 |
| pMB2_00129 | specific | 225322 | 1 | 186 | 3.77888e-46 | 152.135 | COG2602 | YbxI | N | cl21491 |
| pMB2_00130 | specific | 100040 | 55 | 147 | 1.16055e-43 | 141.913 | cd03349 | LbH_XAT | C | cl00160 |
| pMB2_00131 | specific | 225853 | 1 | 214 | 1.78714e-71 | 217.006 | COG3316 | Rve | NA | cl26089 |
| pMB2_00132 | specific | 308240 | 23 | 263 | 1.53996e-81 | 245.177 | pfam02522 | Antibiotic_NAT | NA | cl01051 |
| pMB2_00133 | superfamily | 330919 | 2 | 118 | 0.000635572 | 38.036 | cl26098 | AAA_33 superfamily | C | NA |
| pMB2_00134 | specific | 225511 | 1 | 93 | 6.55569e-10 | 51.8469 | COG2963 | InsE | C | cl26146 |
| pMB2_00135 | superfamily | 331054 | 17 | 247 | 1.54697e-46 | 156.949 | cl26233 | HTH_21 superfamily | NA | NA |
| pMB2_00136 | specific | 225853 | 1 | 214 | 1.78714e-71 | 217.006 | COG3316 | Rve | NA | cl26089 |
| pMB2_00137 | superfamily | 326372 | 434 | 666 | 4.25375e-103 | 319.056 | cl14901 | DDE_Tnp_Tn3 superfamily | C | NA |
| pMB2_00140 | superfamily | 328749 | 36 | 281 | 2.13708e-70 | 218.954 | cl21491 | Transpeptidase superfamily | NA | NA |
| pMB2_00142 | superfamily | 316242 | 4 | 419 | 1.47131e-51 | 178.553 | cl20495 | DDE_Tnp_1_4 superfamily | NA | NA |
| pMB2_00143 | superfamily | 316241 | 5 | 68 | 6.62916e-25 | 89.9198 | cl18663 | DUF4158 superfamily | C | NA |

NA stands for no information available.
